# Phyllosphere microbiome responses to spray-induced gene silencing targeting *Phytophthora infestans* in potato

**DOI:** 10.1038/s41522-026-01040-5

**Published:** 2026-06-15

**Authors:** Samrat Ghosh, Poorva Sundararajan, Bekele Gelena Kelbessa, Farideh Ghadamgahi, Stephen C. Whisson, Mukesh Dubey, Aakash Chawade, Ramesh R. Vetukuri

**Affiliations:** 1 Department of Plant Breeding, Swedish University of Agricultural Sciences, Alnarp, Sweden; 2 Cell and Molecular Sciences, The James Hutton Institute, Dundee, UK; 3 Department of Forest Mycology and Plant Pathology, Swedish University of Agricultural Sciences, Uppsala, Sweden

**Keywords:** Biotechnology, Microbiology, Molecular biology, Plant sciences

## Abstract

Recent years have seen a growing interest in the use of RNA interference to control filamentous pathogens, creating a new niche in plant disease management. Strategies like spray-induced gene silencing (SIGS) offer effective and environmentally friendly alternatives to chemical disease control but remain underexplored. Given the profound influence of microbiomes on plant health and crop productivity, knowledge of how spraying double-stranded RNAs (dsRNAs) can impact plant microbial communities is needed to facilitate the transition of SIGS from the laboratory to practical large-scale use. We have therefore investigated changes in the bacterial and fungal communities of the phyllosphere in the economically important potato plant after spraying with dsRNA targeting the phytopathogen *Phytophthora infestans*. Spraying with dsRNA alone had little effect on the relative abundance of the dominant species found in the native potato phyllosphere. However, there were small time-dependent changes in the composition of the bacterial communities, and much larger changes in bacterial community metrics were observed after *P. infestans* inoculation. We also observed maintenance of potentially beneficial bacterial genera in dsRNA-treated plants in addition to composition changes linked to the plant’s natural defense response upon *P. infestans* infection. Together, these observations support the view that dsRNA spraying enables safe and targeted pathogen control in potato.

## Introduction

With the rapid growth of the global population and increasing challenges in agricultural food production due to pests and diseases associated with climate change, feeding the world’s current and future population is becoming a major concern^1,2^. For example, potato (*Solanum tuberosum*), the world’s third largest staple crop, faces multiple challenges from pests and diseases^3^, creating a significant threat to global food security^4,5^. The pathogen with the greatest impact on potato production is *Phytophthora infestans*^6^. It causes late blight and poses a constant threat to food security, especially in regions where potato is an important food source^7^. This hemi-biotrophic pathogen infects leaves and tubers, spreads rapidly and results in complete defoliation and tuber rot, potentially destroying an entire field within a few days^8^. Estimates of annual global yield losses and costs associated with *P. infestans* control vary, but several studies report losses of 3–10 billion USD per annum^4,9^. Moreover, yield losses due to inadequate control of *P. infestans* are higher in developing countries than in developed ones^10^.

To combat the devastating effects of late blight, potato growers rely on resistant varieties and intensive spraying with preventative and systemic synthetic crop protection mixtures^11^. Unfortunately, disease control through host resistance has limited success due to genetic variation in *P. infestans* and the presence of genes encoding effector proteins in dynamic regions of the *P. infestans* genome, which enable the pathogen to rapidly evolve and overcome host resistance genes^12–14^. Moreover, chemical crop protection agents can have negative impacts on human health and the environment, and are becoming less effective as strains with reduced sensitivity emerge^15,16^. A further complication is that legal restrictions and public concern about the intensive use of chemical pesticides have limited chemical control of plant pathogens, including *P. infestans*. For example, in 2022 the European Union (EU) introduced the European Green Deal, which calls for a 50% reduction in the use and risks of chemical pesticides by 2030^17^. This announcement, combined with increasingly strict regulations, has reduced the usage of some chemical pesticides approved for plant disease control, creating a demand for non-chemical alternatives and integrated pest management approaches.

RNA interference (RNAi) technologies have recently drawn attention for their potential in sustainable crop protection. One such technology is spray-induced gene silencing (SIGS)^18^, which uses exogenous double-stranded RNA (dsRNA) to silence target genes in plant pathogens. RNAi is a master regulatory mechanism that exists in most eukaryotic organisms and has multiple functions, including the repression of target gene expression at the transcriptional or post-transcriptional levels by small RNAs (sRNAs) derived from long dsRNAs^19,20^. Silencing of specific pathogen genes using SIGS can significantly impair host infection without altering the plant genome. For example, SIGS reduced *Fusarium graminearum* infection in barley^18^, *Botrytis cinerea* infection in tomatoes and strawberries^21^, and *P. infestans* infection in potato leaves^22^.

Despite these promising results, little is known about the broader effects of spraying dsRNA for disease control, particularly its impact on plant-associated microbes that colonize the leaf surface and play key roles in disease control, nutrient cycling, abiotic stress tolerance, and promotion of plant growth^23,24^. Previously, we found that spraying dsRNA can alter the structural composition and diversity of the bacterial microbiota of wheat and barley phyllospheres but not the fungal microbiota, and that the nature and magnitude of these changes depend on the choice of dsRNA and the host studied^25^. Building on this, the current study evaluates the impact of spraying dsRNA targeting *PiGPB1* (PITG_06376, essential for sporangial formation and growth) and *PiCut3* (PITG_12361, a cutinase vital for host cell penetration and establishment of infection) genes in *P. infestans* on the phyllosphere microbiome of potato.

## Methods

### Plant and *P. infestans* material

Four-week-old potato plantlets (cultivar Désirée) grown in tissue culture media (4.4 g of Murashige-Skoog medium (Duchefa), 10 g of sucrose, 0.5 g of 2-(N-morpholino) ethanesulfonic acid (MES) and 7.5 g of plant agar dissolved in 1 l of distilled water, pH 5.8) were transplanted into 2.5 l pots filled with well-draining fertilized compost. The plants were grown under controlled climatic conditions with day/night temperatures of 20/19 °C, 60% relative humidity, and a day/night cycle featuring 16 h of 200 µmol/m^2^/s daylight with 8 hours of darkness. *P. infestans* 88069 was grown on rye agar in Petri plates and incubated at 20 °C for two weeks to obtain sporangia^26^. The sporangia were collected in sterile water by gently scraping the surface using an L-spreader and passing the spore suspension through a 40-micron filter. The sporangial concentration was calculated using a Fuchs-Rosenthal chamber and adjusted to 50,000 sporangia/ml before plant inoculation.

### In-vitro dsRNA synthesis

*P. infestans* RNA was extracted from seven-day-old mycelia using the RNeasy Plant Mini kit (Qiagen). One microgram of RNA was used for first-strand synthesis with the iScript cDNA Synthesis kit (Bio-Rad). In-silico primers containing the T7 promoter sequence were designed using NCBI Primer-BLAST for both of the dsRNA targets in *P. infestans* (Table 1)^27^. Polymerase chain reactions were performed with *P. infestans* cDNA and the dsRNA-specific T7 primers using Phusion polymerase (ThermoFisher Scientific), applying the reaction conditions recommended by the manufacturer. The PCR products were purified using the QIAquick PCR Purification kit (Qiagen) before proceeding with in-vitro transcription. DsRNA was synthesized using the MEGAscript RNAi Kit (Invitrogen), with the appropriate PCR-amplified products as templates. In addition, the control template provided with the kit was used to synthesize non-specific dsRNA (Nsp) to serve as a control in subsequent experiments.

**Table 1:** dsRNA synthesis and amplicon sequencing primers

Primer type	Primer name	Primer sequence
In-vitro dsRNA synthesis	T7 *PiGPB1* FP	GTAATACGACTCACTATAGGGCTCTACGCTCCAGTTGGGTC
	T7 *PiGPB1* RP	GTAATACGACTCACTATAGGGTAGATATGCGCTCCGGAAGT
	T7 *PiCut3* FP	GTAATACGACTCACTATAGGCAACCACGTCGTGTCTATCG
	T7 *PiCut3* RP	GTAATACGACTCACTATAGGCTAAGGCAGCAGCTTTCTCG
Amplicon sequencing	Bac_799F	AACMGGATTAGATACCCKG
	Bac_1115R	AGGGTTGCGCTCGTTG
	Fun_ITS1Kyo2F	TAGAGGAAGTAAAAGTCGTAA
	Fun_ITS86R	TTCAAAGATTCGATGATTCA
	Oom_ITS1oo	GGAAGGATCATTACCACAC
	Oom_ITS3oo	AGTATGYYTGTATCAGTGTC
	Oom_ITS4	TCCTCCGCTTATTGATATGC

### Plant assay: dsRNA treatment*, P. infestans* inoculation, and sample collection

Leaves from the same levels of development from four-week-old potato plants were sprayed with two ml (10 µg) of a single dsRNA type (dsRNA *PiGPB1*/dsRNA *PiCut3*) per plant using an airbrush (CoCraft) and compressor (Biltema). Untreated plants (NT), water-sprayed plants (W), and plants sprayed with 10 µg (in two ml) of non-specific dsRNA (Nsp) were included as experimental controls. Leaf samples without *P. infestans* inoculation were collected 24, 48, and 72 h after spraying. For the inoculated samples, plants were drop-inoculated with 15 µl of 50,000 *P. infestans* sporangia/ml twenty-four hours after spraying with dsRNA. Inoculated leaf samples were collected at 48 and 72 h after spraying. Samples were collected using three leaf punches per sample (diameter: 10.8 mm). Three biological replicates were collected for each treatment. The collected samples were stored at −80 °C until needed for DNA extraction.

### DNA extraction

DNA was extracted from the potato leaf samples using the DNeasy PowerSoil Pro kit (Qiagen). Frozen samples were ground to a fine powder in a pre-chilled mortar and pestle filled with liquid nitrogen. DNA was then extracted following the manufacturer’s suggested protocol, with an additional modification in the washing step. Washing with solution EA was repeated three times to ensure complete removal of phenolic compounds.

### Amplicon sequencing

Extracted DNA was sent for amplicon sequencing (LGC Genomics). The 799F-1115R primer pair targeting the 16S rRNA gene was used for bacteria, while the ITS1Kyo2F-ITS86R and semi-nested ITS1oo/ITS3oo-ITS4 primers targeting the ITS gene sequences were used for fungi and oomycetes, respectively^28–30^. The PCR reactions were performed using 1–10 ng of extracted DNA and 15 pmol of the appropriate forward and reverse primers (Table 1) in a 20 μL volume of 1 x MyTaq buffer containing 1.5 units of MyTaq DNA polymerase (Bioline GmbH, Luckenwalde, Germany), and 2 μl of BioStabII PCR Enhancer (Sigma-Aldrich Co.). All forward and reverse primers contained the same 10 nt barcode sequence. PCRs were performed for 30–40 cycles for bacterial and fungal primers (30–33 cycles for 799F-1115R and 35–40 cycles for ITS1Kyo2F-ITS86R) using the following parameters: pre-denaturation at 96 °C for 1 min, denaturation at 96 °C for 15 s, annealing at 55 °C for 30 s and extension at 70 °C for 90 s. Oomycete DNA was amplified using nested PCR with a pre-amplification step followed by a two-step PCR. The pre-amplification PCR ran for only 15 cycles with an annealing temperature of 48 °C. The first amplification of the two-step PCR lasted for 20 cycles of 1 min pre-denaturation at 96 °C followed by denaturation for 15 s at 96 °C, annealing for 30 s at 58 °C, and extension for 90 s at 70 °C with a final hold at 8 °C. The second amplification of the two-step PCR was identical to the first step but with a modified annealing step of 3 cycles at 50 °C followed by 7 cycles at 58 °C.

The DNA concentration of the amplicons was assessed by gel electrophoresis. About 20 ng of indexed amplicon DNA from each sample was pooled, and the pools were purified using one volume of Agencourt AMPure XP beads (Beckman Coulter, Inc., IN, USA) to remove primer dimers and other small mispriming products. Further purification was performed with MiniElute columns (QIAGEN GmbH, Hilden, Germany). Illumina libraries (Illumina, Inc., CA, USA) were constructed from the purified amplicon pool DNA (100 ng each) using the Ovation Rapid DR Multiplex System 1–96 (NuGEN Technologies, Inc., CA, USA). Library DNA was then pooled, size selected by preparative gel electrophoresis, and sequenced on an Illumina MiSeq using V3 Chemistry (2 × 300 bp).

### Meta-barcode processing of bacterial, fungal and oomycete communities

Adapter sequences were filtered out from Illumina FASTQ reads using fastp v0.24.1^31^. Trimmed bacterial 16S rRNA, fungal ITS, and oomycete ITS sequence data were then processed using QIIME2 (version qiime2-2023.7)^32^. The QIIME2 cutadapt plugin^33^ was used for primer removal. The DADA2 plugin^34^ was employed to identify unique DNA sequences, called amplicon sequence variants (ASVs). For taxonomic assignments, filtered ASVs were trained against qiime2 compatible SILVA v138.1 (bacteria), UNITE v9.0 (fungi), and customised (oomycetes) reference databases with a naive Bayes classifier^35,36^. Unassigned ASVs and ASVs assigned to chloroplasts or mitochondria were removed.

### Community composition and diversity analysis

The microbiome data (16S and ITS) were analysed in R v4.4.2^37^. For downstream analysis, a phyloseq object was created for each dataset (bacteria, fungi, and oomycetes) using R package Phyloseq v1.50.0^38^. Rarefaction curves were drawn to assess sample quality. Core microbiome data were plotted with the plot_core() function (R package microbiome v1.30.0)^39^. Relative abundance was plotted with the plot_taxa_composition() function (R package microbiomeutilities v1.00.17)^40^.

The normality (*p* < 0.05) of the data was checked, and statistical tests were performed accordingly. The “Simpson Index” was used to measure the alpha diversity (R package vegan v.2.6-8) of each sample^41^. A parametric one-way Analysis of Variance (ANOVA) followed by pairwise t-tests was also conducted, applying a significance threshold of *p* < 0.05.

A permutational multivariate analysis of variance (PERMANOVA/ADONIS) with 999 permutations (R package vegan v.2.6-8) was performed to evaluate the diversity of different groups^41^. Bray-Curtis dissimilarity (R package vegan v.2.6-8) was used for principal coordinates analysis (PCoA)^41^.

Analysis of Compositions of Microbiomes with Bias Correction (ANCOM-BC) was used for analysing differential abundance between groups. It was implemented through the R package ANCOMBC v1.6.4^42^, with q values below 0.05 considered significant.

### Co-occurrence network construction

Network construction was performed using the R package SpiecEasi v1.1.1^43^ to address compositionality bias. The Fruchterman-Reingold (FR) layout algorithm was utilized for network visualization. Network modules were delineated with the cluster_fast_greedy and walk_trap algorithms. Network properties were computed with igraph v2.1.4. ZiPi plots were generated using the ZiPiPlot() function of ggClusterNet v1.00^44^. Network properties were classified into four groups (i) network hubs (Zi > = 2.5 and Pi > = 0.62), (ii) module hubs (Zi > = 2.5 and Pi < 0.62), (iii) connectors (Zi < 2.5 and Pi > = 0.62), and (iv) peripheral nodes (Zi < 2.5 and Pi < 0.62).

## Results

### Overall counts and taxonomy assignments

To investigate the potential effects of dsRNA spraying on the microbial communities of potato leaves, a pot experiment was conducted wherein potato plants were sprayed with either controls (no treatment/NT or water treatment/W) or the individual dsRNAs (Nsp, GPB1, Cut3). Amplicon sequencing of leaf samples produced a total of 14,090,652 raw paired-end reads (5,980,756 bacterial, 4,904,556 fungal, and 3,205,340 from oomycetes) from 178 samples (75 each for bacteria and fungi and 28 for oomycetes). The average number of reads per sample was 79,743 ± 33,550 (mean ± SD) for bacteria, 65,394 ± 28,468 for fungi, and 221,057 ± 39,326 for oomycetes (Suppl. data S1). After filtering the reads for quality, 19.80% of reads were removed, leaving a total of 11,300,259 quality filtered reads (5,335,430 bacterial, 4,359,296 fungal, and 1,605,533 from oomycetes) with averages of 71,139 ± 29,719, 58,123 ± 25,436, and 110,726 ± 19,875 paired reads per sample, respectively. This generated a total of 3876 Amplicon Sequence Variants (ASVs) (3114 bacterial, 713 fungal, and 49 from oomycetes). Representative sequences from the ASVs were then assigned to 30 different phyla (23 bacterial, 6 fungal, and 1 in oomycetes), of which two were unclassified. A total of 663 genera (473 bacterial, 188 fungal, and 2 in oomycetes) were found, of which 74 (11.16%) were unclassified (Suppl. data S2, S3, S4). The rarefaction curves of all the sequenced samples displayed a plateau, indicating that the sampling provided sufficient diversity and coverage for the tested conditions. When discussing the results, treatments including *P. infestans* inoculation carry the suffix “+ Pi”. The treatments apart from Nsp and Nsp + Pi were also grouped into no dsRNA (ND: NT and W), no dsRNA with inoculation (ND + Pi: NT + Pi and W + Pi), dsRNA (GPB1 and Cut3), and dsRNA with inoculation (dsRNA + Pi: GPB1 + Pi and Cut3 + Pi) in order to draw grouped inferences.

### Bacterial community characteristics

**Fig. 1:**
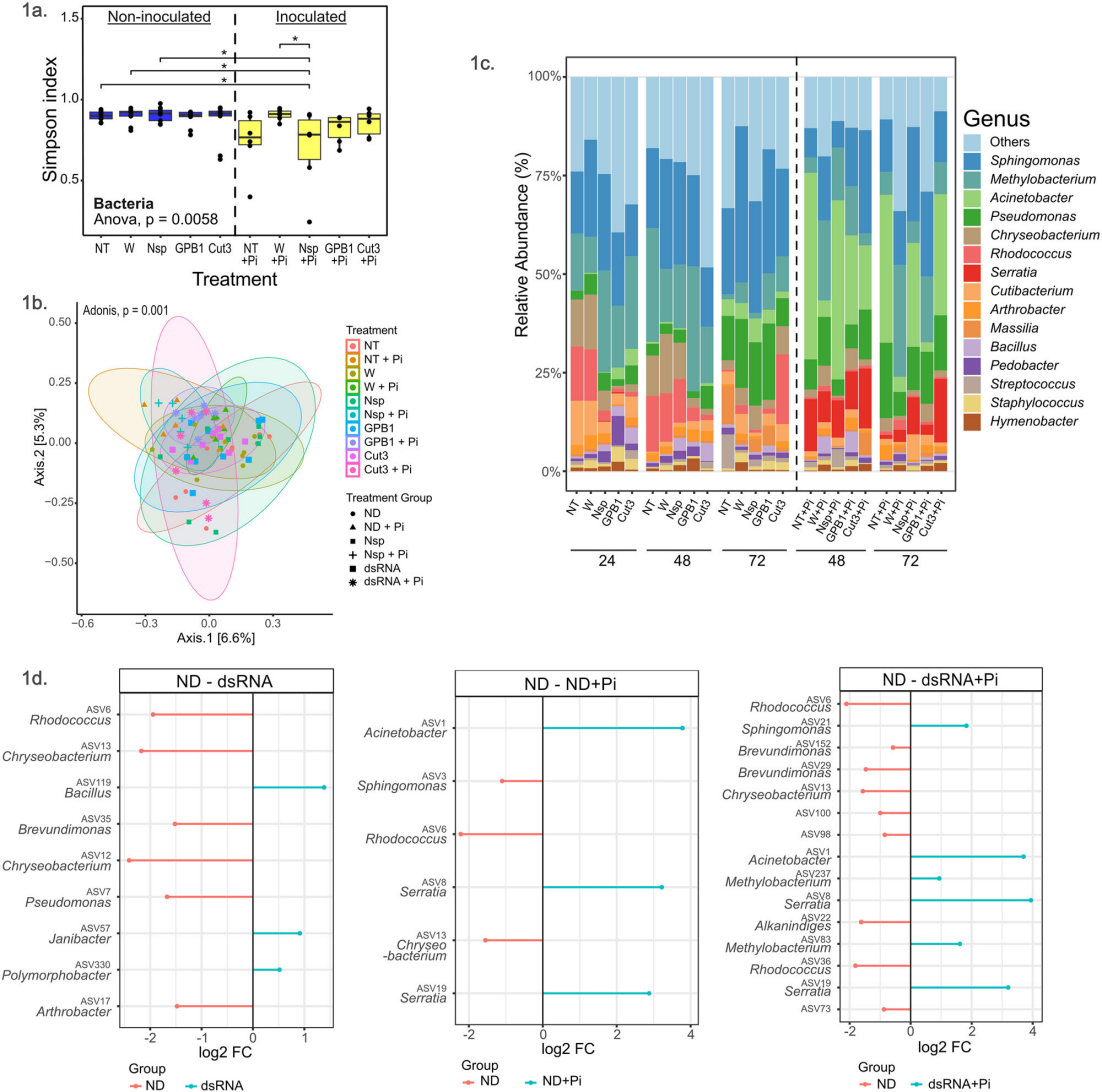
Bacterial community characteristics following dsRNA spray. **a** Boxplots showing alpha-diversity across treatments, calculated using the Simpson index. Asterisks indicate *p* < 0.05 (Student’s t-test). **b** PCoA plots showing the beta-diversity based on the Bray-Curtis dissimilarity distance. The colours correspond to treatments and the shapes to treatment groups. The ellipses represent a 95% confidence interval. The diversity of the groups was assessed using PERMANOVA with Adonis. **c** Community composition represented as the relative abundance of the 15 most abundant genera. **d** ANCOM-BC results showing the differential abundance of bacterial ASVs between groups. Only ASVs showing significant (*q* < 0.05) fold changes are shown.

Diversity metrics for the microbial communities in the leaves were calculated using the Simpson index (alpha-diversity) and the Bray-Curtis dissimilarity distance (beta-diversity). The tested conditions had a significant effect on the richness of the bacterial communities (Simpson, ANOVA, *p* = 0.0058) (Fig. 1a). However, further pairwise comparisons showed that the individual dsRNA treatments (GPB1 and Cut3) were not statistically significant from the control treatments (NT, W) (Student’s t-test, *p* > 0.05), although Nsp + Pi did differ significantly from the NT, W, W + Pi, and Nsp treatments (*p* < 0.05) (Suppl. data S5).

The beta-diversity, representing the compositional similarities and community shifts between the bacterial communities, revealed large overlaps between the control and dsRNA treatments (Fig. 1b). We also looked at shifts between different sampling times. At 24 h post spray, the ND, Nsp, and dsRNA samples exhibited visual separation, while at 48 h after spraying the dsRNA Cut3 samples (Cut3 and Cut3 + Pi) clustered separately in both inoculated and non-inoculated samples (Suppl. Fig. 1a). However, pairwise comparisons revealed no significant differences between the treatments (Suppl. data S6).

The relative abundance of the observed ASVs was then plotted to reveal differences in bacterial community composition between treatments (Fig. 1c, Suppl. data S7). The dominant genera in non-inoculated samples were *Sphingomonas* (16.51–30.42%), *Pseudomonas* (5.87–8.53%), and *Methylobacterium* (8.88–18.80%), which accounted for 38–51% of the total relative abundance. Conversely, the dominant genera of inoculated samples were *Acinetobacter* (3.79–42.25%), *Sphingomonas* (9.79–19.28%), *Methylobacterium* (5.91–23.91%), *Pseudomonas* (7.53–13.82%), and *Serratia* (5.91–15.18%), which accounted for 58–78% of the total relative abundance. In both control and dsRNA samples, the bacterial community composition under both inoculated and non-inoculated conditions was characterized by the presence of common and highly-abundant ASVs, with some genera becoming more abundant upon *P. infestans* inoculation. The abundances of genera such as *Massilia*, *Acinetobacter*, *Pseudomonas*, and *Serratia* changed over time in all treatments.

To better understand these compositional changes, the differential abundances of individual ASVs across treatments were analysed using ANCOM-BC (Fig. 1d). Overall, more ASVs were depleted than enriched, and multiple ASVs belonging to the same genera were found. *Bacillus*, *Janibacter*, and *Polymorphobacter* were more abundant in dsRNA samples than in ND. Conversely, *Rhodococcus*, *Chryseobacterium*, *Brevundimonas*, *Pseudomonas*, and *Arthrobacter* were depleted in dsRNA samples. No taxa exhibited differential abundance between Nsp and ND samples (Suppl. data S8). *Serratia* and *Acinetobacter* were substantially more abundant in all inoculated samples (ND + Pi, Nsp + Pi, dsRNA + Pi) than in ND (Fig. 1d and Suppl. Fig. 1b). Finally, when comparing the dsRNA + Pi treatment to ND, *Sphingomonas* and *Methylobacterium* showed increased abundance, while *Rhodococcus*, *Brevundimonas*, *Chryseobacterium*, *Alkanindiges*, and three unclassified ASVs showed reduced abundance.

### Fungal community characteristics

**Fig. 2:**
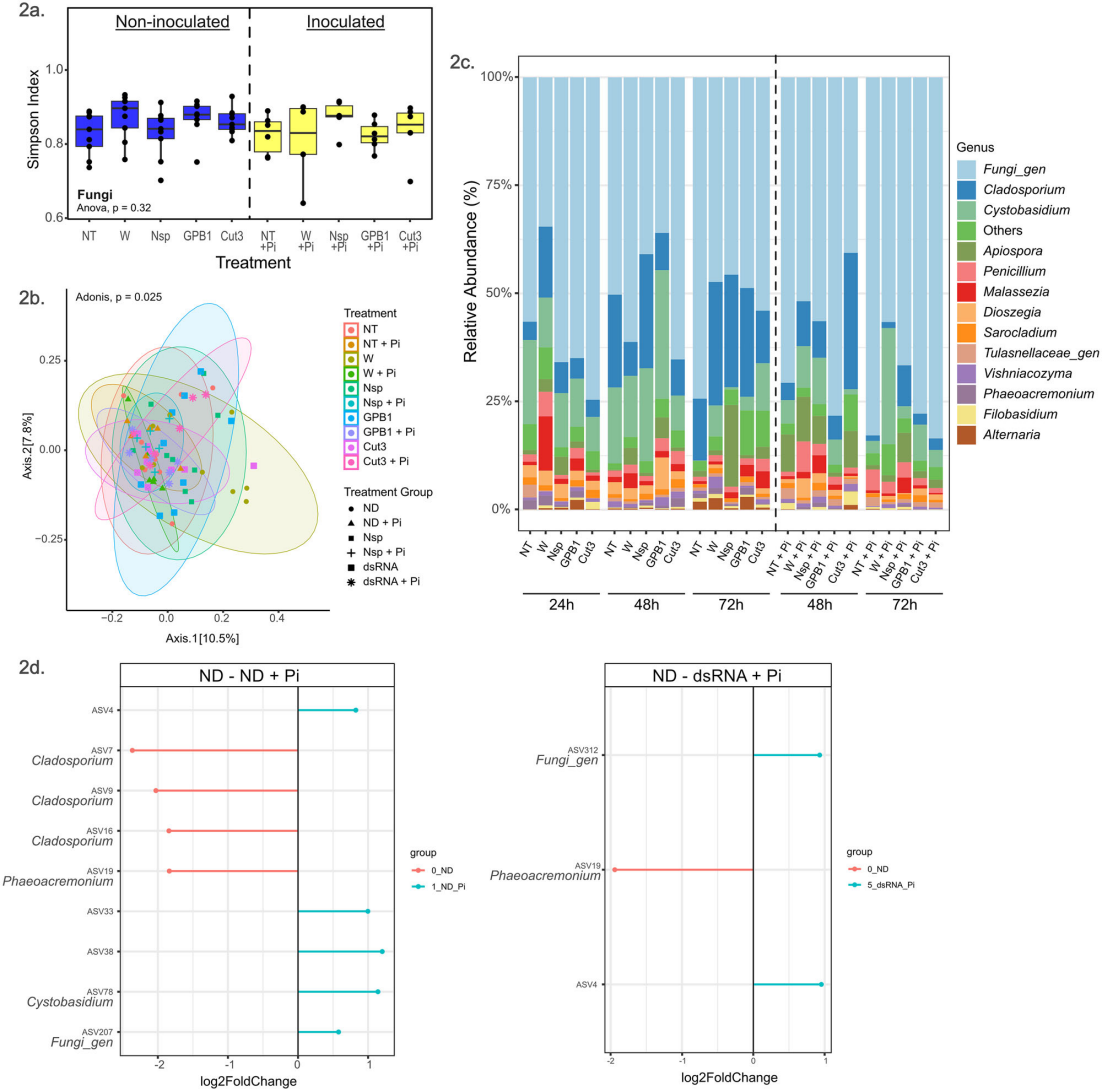
Fungal community characteristics following dsRNA spray. **a** Boxplots showing alpha-diversity across treatments, calculated using the Simpson index. Analysis of variance (ANOVA) and pairwise t-tests showed no significant differences between the groups. **b** PCoA plots showing the beta-diversity based on the Bray-Curtis dissimilarity distance. The colours correspond to treatment and the shapes to treatment groups. The ellipses represent a 95% confidence interval. The diversity of the groups was assessed using PERMANOVA with Adonis. **c** Community composition represented as the relative abundance of 15 most abundant genera. **d** ANCOM-BC results showing the differential abundance of fungal ASVs between groups. Only ASVs showing significant (*q* < 0.05) fold changes are shown.

The alpha-diversity metrics of the fungal communities in the control and dsRNA treatments were comparable (Fig. 2a). There were no significant differences between the control and dsRNA treatments or between the non-inoculated and inoculated conditions (Suppl. data S5).

PCoA plots representing the dissimilarities in the fungal ASVs revealed no distinct clustering of the treatment groups, and there were large overlaps between the controls and dsRNA treatments, with and without inoculation (Fig. 2b). In addition, there were no appreciable differences between the treatments at any of the individual sampling times (Supplementary data S6).

The relative abundance of the top 15 observed taxa showed that the same genera were abundant in all samples, and the fungal composition did not vary greatly between treatments (Fig. 2c, Supplementary data S9). The abundance of genera such as *Cladosporium* and *Cystobasidium* changed over time, irrespective of dsRNA treatment. Further differential abundance analysis using ANCOM-BC identified ND + Pi and dsRNA + Pi as the only treatment groups to have ASVs exhibiting appreciable differential abundance compared to ND (Fig. 2d, Suppl. data S8). The genera *Cladosporium* and *Phaeoacremonium* were depleted in ND + Pi samples, whereas only *Phaeoacremonium* was depleted in dsRNA + Pi samples. Additionally, a few unclassified ASVs were enriched in both inoculated sample groups, while an ASV belonging to *Cystobasidium* was enriched only in ND + Pi samples.

In addition to the fungal reads, sequence data for the ITS gene regions were analysed to detect oomycete reads. Although a large number of oomycete reads were detected, the number of taxa recovered from these reads was too low to make any robust inferences (Suppl. data S10). The oomycete genera identified included *Phytophthora* (99.53–100%) and *Pythium* (0 – 0.46%).

### Complexity and stability of bacterial and fungal community networks

**Fig. 3:**
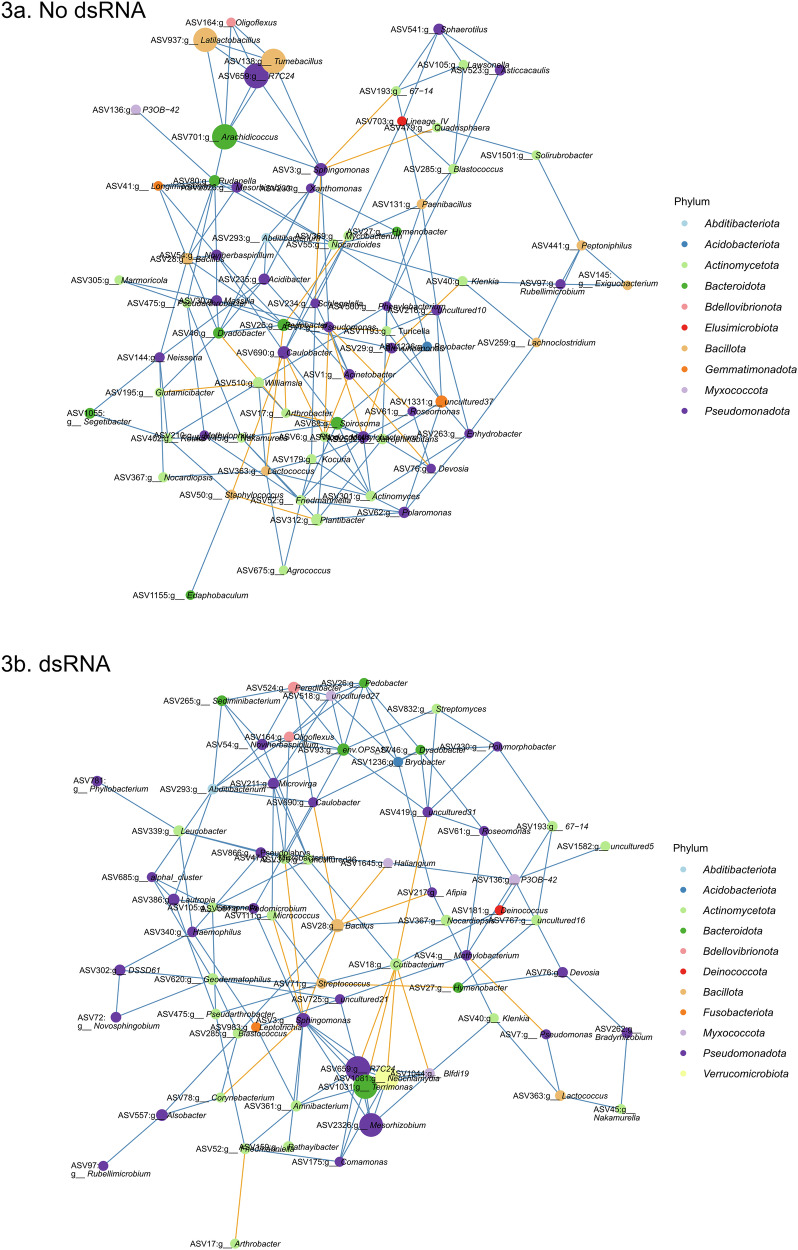
SPIEC-EASI-based bacterial networks. Fruchterman-Reingold (FR) layout implemented for network visualization. Nodes (circles) represent bacterial ASVs and edges (lines) represent positive and negative interactions (dark blue and orange, respectively). Node size, based on degree and colours, represent the phyla the ASVs belong to. Most of the nodes belonged to *Pseudomonadota*, *Actinomycetota* and *Bacteroidota*. **a** Bacterial networks in the ND (no dsRNA) samples. **b** Bacterial networks in the dsRNA samples.

**Fig. 4:**
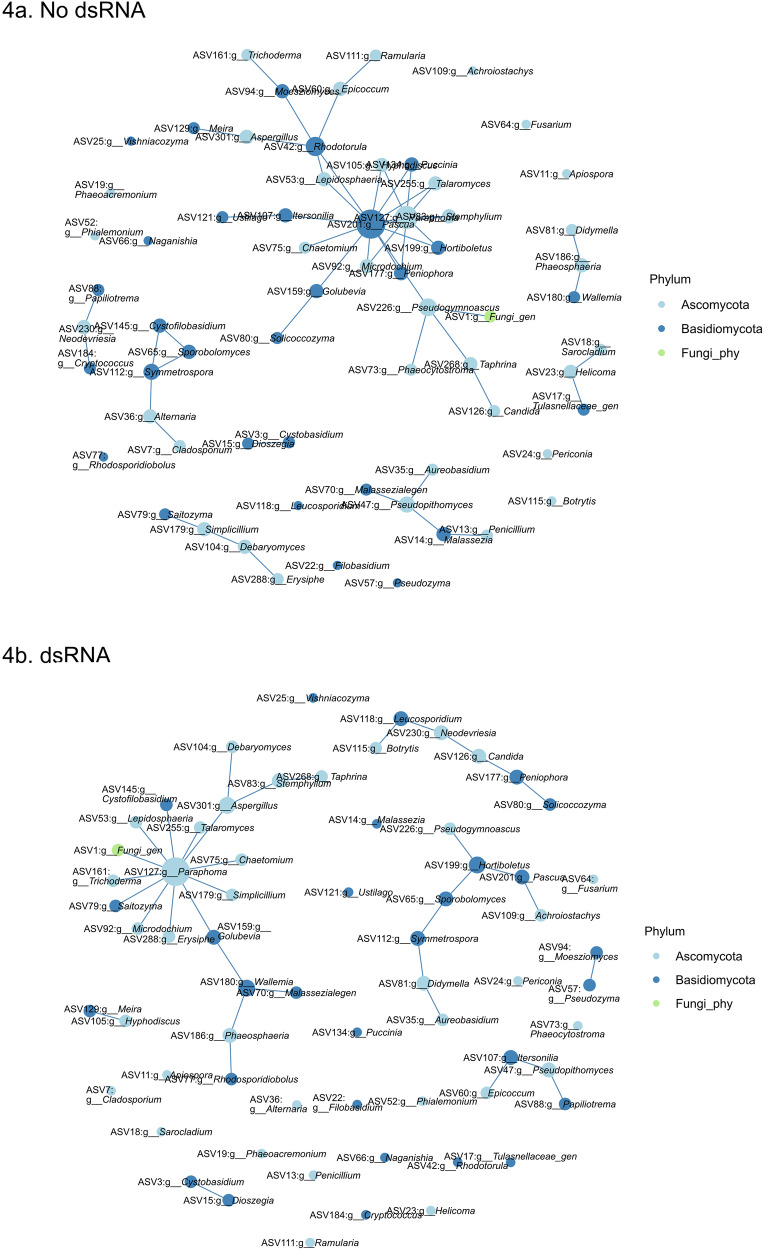
SPIEC-EASI-based fungal networks. Fruchterman-Reingold (FR) layout implemented for network visualization. Nodes (circles) represent fungal ASVs and edges (dark blue lines) represent positive interactions. Node size, based on degree and colours, represent the phyla the ASVs belong to. All nodes belonged to Ascomycota, Basidiomycota or an unknown fungal phylum. **a** Fungal networks in the ND (no dsRNA) samples. **b** Fungal networks in the dsRNA samples.

The bacterial network inferred with SPIEC-EASI (estimated using Meinshausen-Buhlmann’s neighbourhood selection) for the ND treatment contained 240 nodes with 691 edges, while that for the dsRNA treatments contained 240 nodes with 665 edges. In comparison, the fungal networks for the ND and dsRNA treatments both had only 65 nodes, with 53 and 37 edges, respectively (Table 2). Overall, taxa tended to co-occur (positive edges, dark blue lines) rather than co-exclude (negative edges, orange lines). 96.52%/96.99% (ND/dsRNA) of the edges were positive in the bacterial networks while 100% were positive in the fungal networks (Figs. 3, 4, Suppl. data S11).

**Table 2:** Topological features of SPIEC-EASI-based bacterial and fungal networks

Topological features	Bacterial networks		Fungal networks	
	ND	dsRNA	ND	dsRNA
Total Nodes	240	240	65	65
Total Edges	691	665	53	37
Number of Positive Edges	667 (96.52%)	645 (96.99%)	53 (100%)	37 (100%)
Number of Negative Edges	24 (3.47%)	20 (3%)	0 (0%)	0 (0%)
Average node degree	5.76	5.54	1.63	1.14
Average path distance	4.03	3.95	2.74	2.75
Modularity (Fast greedy/Walk trap)	0.62/0.60	0.62/0.59	0.68/0.68	0.77/0.76

Nodes in the bacterial networks mainly corresponded to the phyla *Pseudomonadota*, *Bacillota*, *Bacteroidota*, and *Actinomycetota*, while those in the fungal networks corresponded to Ascomycota and Basidiomycota. The average node degrees in the control and dsRNA treatments were 5.76 and 5.54 in the bacterial networks and 1.63 and 1.14 in the fungal networks (Table 2). The average path distances in the control and dsRNA treatments were 4.03 and 3.95, respectively, for bacteria, while those for fungi were 2.74 and 2.75, respectively (Table 2).

A network module is defined as a group of ASVs that are tightly linked together. The entire bacterial and fungal networks under both ND and dsRNA treatments were parsed into several modules. The modularity scores (Fast greedy/Walk trap) of the bacterial networks for the ND and dsRNA treatments were 0.62/0.60 and 0.62/0.59, respectively, while the corresponding scores for the fungal networks were 0.68/0.68 and 0.77/0.76, respectively (Table 2). The bacterial ND network had a total of 10 modules, with the top three accounting for 22.5%, 15%, and 14.58% of the ASVs. The bacterial dsRNA network had 16 modules, with the top three accounting for 18.33%, 10.41%, and 9.16% of the ASVs (Fig. 3). The total numbers of modules for the ND and dsRNA fungal networks were 11 and 12, respectively, with the top three modules from the respective fungal networks accounting for 21.42%, 14.28% and 11.90% (ND), and 22.58%, 12.90% and 12.90% (dsRNA) of the ASVs (Fig. 4). Interestingly, all 240 bacterial nodes were located inside the modules under all treatments, whereas only 42 out of 53 nodes occurred in nodes within the ND network and only 31 out of 37 nodes occurred inside modules in the dsRNA network.

**Fig. 5:**
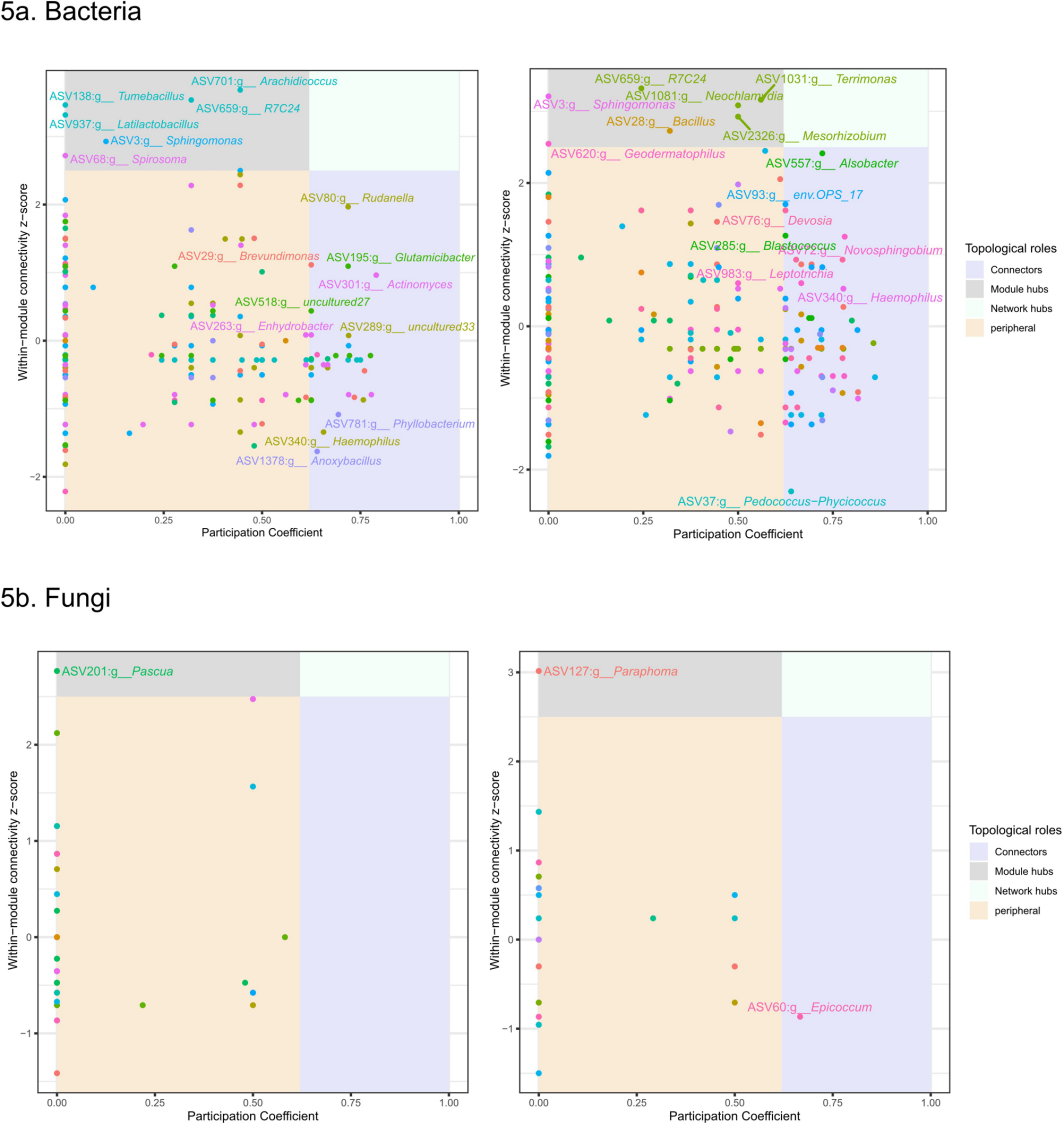
Zi-Pi plots for microbial communities. Plots identifying the roles of different ASVs in the microbial networks based on Zi (between-module connectivity) and Pi (among-module connectivity) scores for individual ASVs. Zi-Pi plots for (**a**), bacterial and (**b**), fungal communities in the ND and dsRNA groups. Peripheral, connector, module hub, and network hub nodes were categorized based on their Zi and Pi scores. The colours of the individual ASVs indicate the modules they belong to.

Plots derived from Zi-Pi analysis showed that 6 ASVs in the ND bacterial co-occurrence network and 7 ASVs in the dsRNA network were keystone or module hub taxa (Fig. 5). Common keystones were ASV3 (g__Sphingomonas) and ASV659 (g__R7C24). The different coloured points represent different modules. Conversely, the fungal ND and dsRNA networks each displayed only one module hub ASV (ASV201 - *Pascua* and ASV127 - *Paraphoma*, respectively). Details of within-module connectivity scores (zi) and among-module connectivity scores/participation coefficients (pi) for each ASV (Module hubs, Network hubs, Connectors, and Peripheral) are provided in (Suppl. data S11).

## Discussion

A diverse array of microorganisms inhabit plants and function in interdependence with them. This microbial consortium constitutes the plant microbiome and confers fitness advantages during plant growth and development, responses to stressors, and defence against diseases^45–48^. The role of microorganisms such as *Pseudomonas*, *Rhizobium*, *Bacillus*, *Methylobacterium*, and several other bacteria and fungi in growth promotion and stress tolerance is well documented^49,50^. In addition, a study on soils exposed to recurrent droughts revealed the development of an ecological memory that can improve resilience during subsequent drought events^51^. Microbial inoculants are also known to confer plants with the ability to develop induced systemic resistance (ISR), a vital plant defense mechanism^45,52^. Both plant-microbiome interactions and interactions between microbial constituents can thus influence yields, shape plant immunity, and drive ecosystem functioning^53–55^.

Research on non-coding RNAs has demonstrated their vast potential for establishing RNA-based strategies as an effective alternative to conventional plant protection methods^56–58^. However, a well-rounded assessment is required to ensure these strategies are also environmentally friendly. To this end, this study has investigated how spraying dsRNA targeting the oomycete pathogen *P. infestans* alters the microbial communities of the potato phyllosphere.

Analysis using various microbial community metrics revealed that dsRNA spraying altered the bacterial communities of the plant phyllosphere. While no significant shifts caused by dsRNA treatment were apparent in the PCoA plots, changes in the community structure at different sampling times indicated an influence of time on bacterial composition. The control and dsRNA treatments clustered separately at the earlier time points, which may reflect the sensitivity of the bacterial communities to an external disturbance^59^. Similar shifts in community composition have been observed in studies on the temporal dynamics of microbial communities subject to environmental perturbations^60^. Further analysis of microbial communities in samples collected several days after dsRNA spraying would be valuable to determine whether the bacterial communities can recover and restore their original compositions.

Beta-diversity, composition, and differential abundance analyses all indicated that larger shifts in the bacterial communities occurred after inoculation with the pathogen rather than after dsRNA spraying. Some species belonging to the genera *Serratia* and *Acinetobacter* are known to exhibit biocontrol activity against *P. infestans*^61^ and other pathogenic *Phytophthora* spp^62–64^. The enrichment of these genera in both ND + Pi and dsRNA + Pi samples may therefore suggest selection for the bacterial taxa that are fittest to survive the presence of infection and the compositional changes triggered by the plant’s response to pathogen invasion. Additionally, *Sphingomonas* and *Methylobacterium* spp., which were selectively enriched in dsRNA + Pi samples, are frequently present in plant phyllospheres, and some species belonging to these genera have been associated with plant growth promotion, biocontrol potential, and disease suppression in several plants including potato^65–69^. Their increased abundance distinctively in dsRNA-treated samples with pathogen inoculation may suggest enrichment of plant beneficial bacteria that can help alleviate pathogen proliferation, possibly through altered microbial composition and changed ecological niches. However, characterization of these identified ASVs at the species level and further validation is necessary to ascertain their exact role and influence in the potato phyllosphere. Together, these results suggest that dsRNA spraying preserves compositional changes that are part of the plant’s natural response to pathogen invasion, while potentially also enriching beneficial bacteria in the phyllosphere.

While considering the differences observed above, it is important to note that the core bacterial microbiota remained the same between the control and dsRNA treatments. Spraying with dsRNA therefore, does not change the bacterial richness and diversity of the potato phyllosphere despite altering the relative abundance of a few highly abundant taxa.

In contrast, the fungal community was largely unaffected by dsRNA spraying. The fungal diversity, community structure and composition were similar across the various treatments and conditions. The absence of differentially abundant taxa when comparing the dsRNA and control treatments showed that the fungal communities remained stable after dsRNA spraying. Furthermore, the abundance changes observed in the inoculated samples showed that fewer fungal taxa are differentially abundant upon *P. infestans* infection compared to bacteria. These findings are consistent with the observation that fungal communities tend to display greater stability than bacterial communities when exposed to a disturbance^70^.

The network analyses showed that the leaf microbial community network remained stable after dsRNA spraying. The assembly of the microbial community is shaped by specific interactions between members of the microbiota including bacteria, fungi, oomycetes, and protists on the leaf surface^71^. Previous reports suggest that 25–30 samples per group is sufficient for network analysis, although there is no universal rule for determining the minimum sample size^72^. The group of samples representing *P. infestans* inoculation was too small to infer a network graph, and as such, the network analysis therefore focused on the response to dsRNA spraying.

Biological networks may feature hundreds or even thousands of nodes. The total number of nodes determines the network size. Larger networks are generally considered to be more stable, with greater resistance to perturbation^73^. The properties of co-occurrence networks can reveal the inherent characters of microbial interactions in response to external stimuli^70^, and network connectivity and strength are crucial for plant resistance against pathogens^74^. However, network stability depends on other metrics, such as robustness. Robust networks are those that can avoid rapid collapse in the aftermath of disturbance. Accordingly, current metrics suggest that greater network robustness corresponds to greater stability^75^.

The concept of keystone or hub taxa was introduced by Paine to describe taxa that are crucial for ecosystem function and stability^76^. The literature suggests that keystone or hub microorganisms are tightly connected and have a positive association with stability^48^. In the phyllosphere, keystone taxa can serve as mediators between the plant and the microbiome, in microbe–microbe interactions, and in biocontrol activity. We identified two bacterial nodes, namely ASV3 (g__Sphingomonas) and ASV659 (g__R7C24), that acted as module hubs under all treatments. This observation is consistent with the results of a recent study showing that *Sphingomonas* sp. recruited by tomato root exudates containing succinic acid enhances resistance to bacterial wilt disease^77^. Other studies have similarly highlighted the role of *Sphingomonas* sp. in biocontrol^78,79^.

The hub microbes (i.e., the nodes with the highest degree) belonged to the bacterial phyla *Pseudomonadota*, *Actinomycetota*, *Bacillota*, *Bacteroidota*, and *Verrucomicrobiota* as well as the fungal phylum Ascomycota. This aligns with earlier reports indicating that *Pseudomonadota*, *Actinomycetota*, and *Ascomycota* are the most abundant microbial phyla in plants and soil^80^, and that dysbiosis in *Actinomycetota* abundance in the tomato rhizosphere causes bacterial wilt disease^81^. Some key taxa that exhibited high node degree and betweenness centrality in an earlier study on the root microbiome^82^, notably *Actinomycetota*, were also identified as hub microbes in this work (Suppl. data S11).

Interestingly, some comparatively rare ASVs were also identified as module hubs and connectors. These findings align with literature reports suggesting that dominant and minor species can sometimes “switch” their roles in response to sudden environmental stimuli^83^. However, it should be taken into account that some of these observations for rare ASVs may also be attributable to artifacts, spurious associations, statistical error, or biases associated with polymerase chain reaction (PCR) and sequencing technology^84^. A further consideration is that one limitation of current network analyses is their inability to support robust statistical comparisons. Another is that relatively large sample sizes are needed to infer biologically or ecologically relevant interactions^43,85^.

This work examined the effects of dsRNA spraying targeting the oomycete pathogen in a dicot plant. However, the observed shifts in bacterial and fungal community metrics were comparable to those seen in an earlier study exploring microbial community changes associated with dsRNA spraying targeting a fungal pathogen in monocot plants^25^. As such, the results presented herein expand current knowledge about the effects of spraying dsRNA on the phyllosphere microbiome of plants and confirm our initial hypothesis concerning the overall safety and target specificity of SIGS.

The use of non-coding RNAs, including dsRNAs and sRNAs for plant protection, has increased in recent years. Effective use of their potential for disease control through SIGS will require a deep understanding of their effects on host plants and their microbiomes. This study has shown that dsRNA spraying does not significantly change the diversity of the bacterial and fungal communities in the potato phyllosphere. However, the bacterial communities exhibit modest changes in composition in a time-dependent manner, as well as larger changes in composition driven by *P. infestans* inoculation. Network analyses similarly indicated that dsRNA spraying had no significant impact on microbial community assembly. These results support our initial hypothesis about the safety and target specificity of dsRNA spraying. Extensive research has shown that plant microbiomes are influenced by geographical location, host plant variety, pollutants, seasonal variation, and other factors. It would therefore be desirable to complement the work presented herein with field-level studies that include multiple plant genotypes and a wider range of sampling times to obtain a broader picture of the intricate mechanisms shaping microbial communities during SIGS.

## Supplementary information

Supplementary figures

Supplementary data S1

Supplementary data S2

Supplementary data S3

Supplementary data S4

Supplementary data S5

Supplementary data S6

Supplementary data S7

Supplementary data S8

Supplementary data S9

Supplementary data S10

Supplementary data S11

Supplementary data S12

## Data Availability

The amplicon data used in this project are deposited in the Sequence Read Archive database under BioProject accession no. **PRJNA1060527**.

## Code availability

All the scripts/codes used for the data analysis are available in GitHub (https://github.com/samratencode/amplicon_analysis).
