# Supplementary material for: Phyllosphere microbiome responses to spray-induced gene silencing targeting Phytophthora infestans in potato
Source: NPJ Biofilms Microbiomes. 2026 Jun 15;12:118. doi: 10.1038/s41522-026-01040-5 (PMC13270030; doi:10.1038/s41522-026-01040-5)
Supplement: Supplementary file 1 — Supplementary figures [file 41522_2026_1040_MOESM1_ESM.pdf]

S1a. Time point-specific bacterial beta-diversity

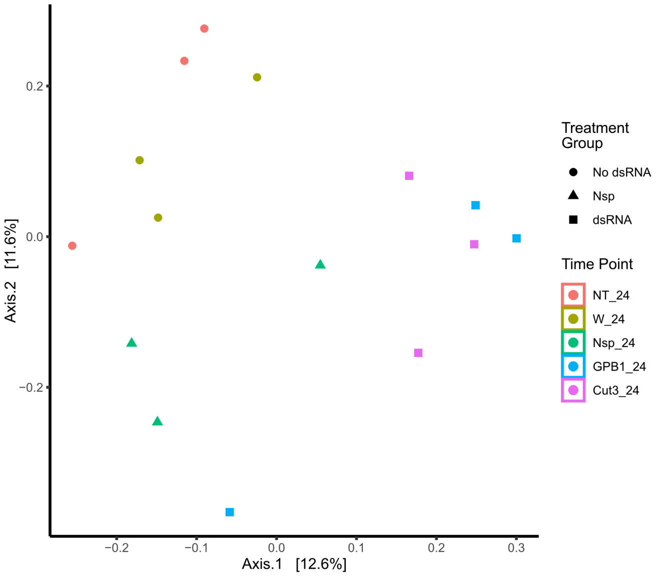

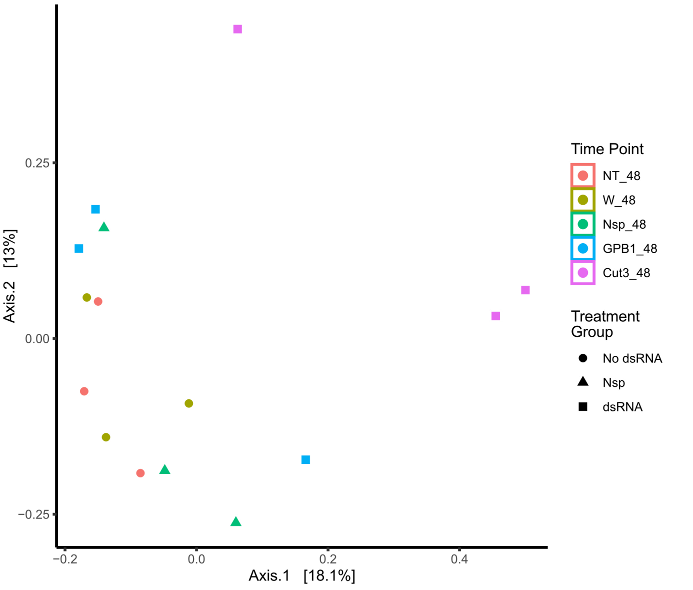

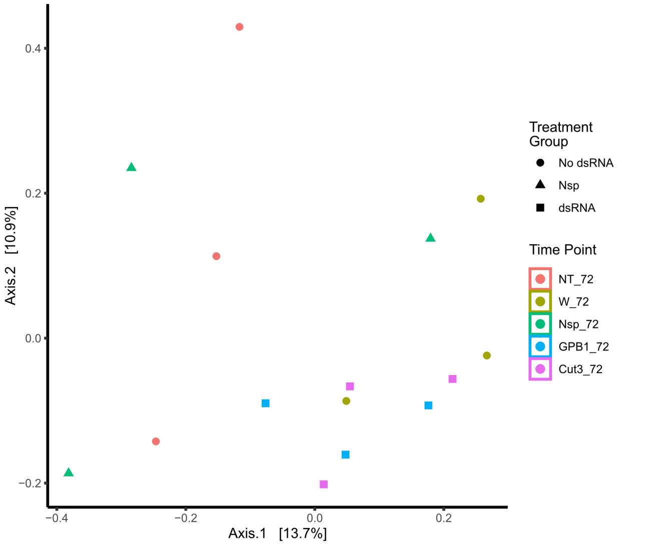

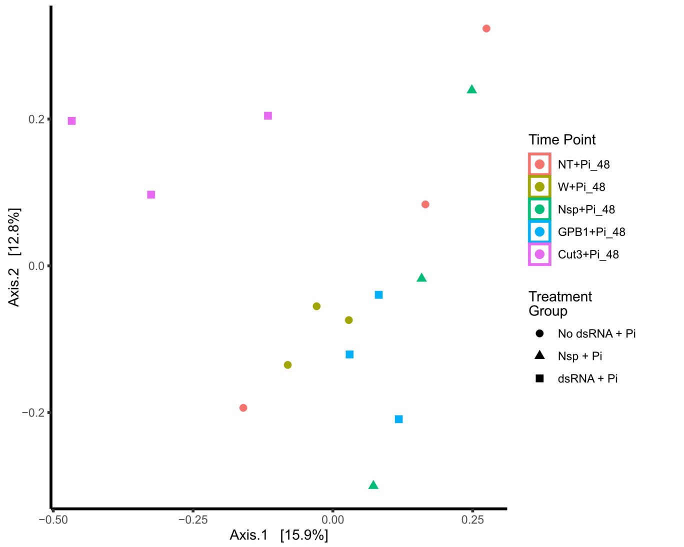

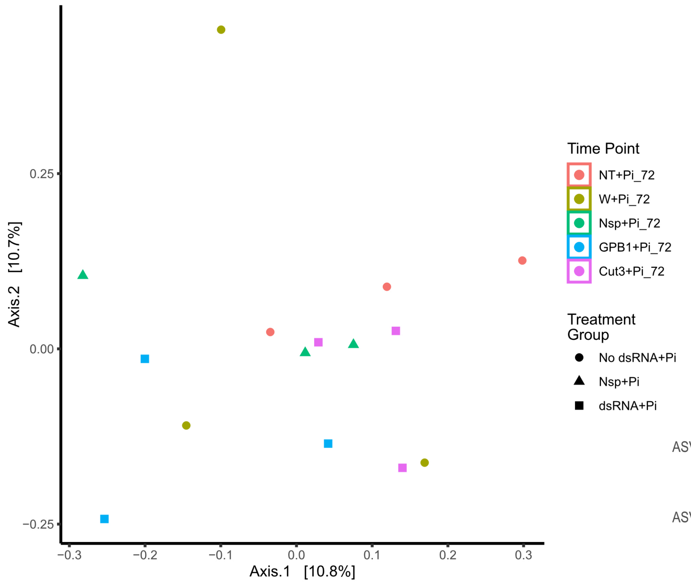

S1b. ANCOM-BC - ND vs. Nsp + Pi

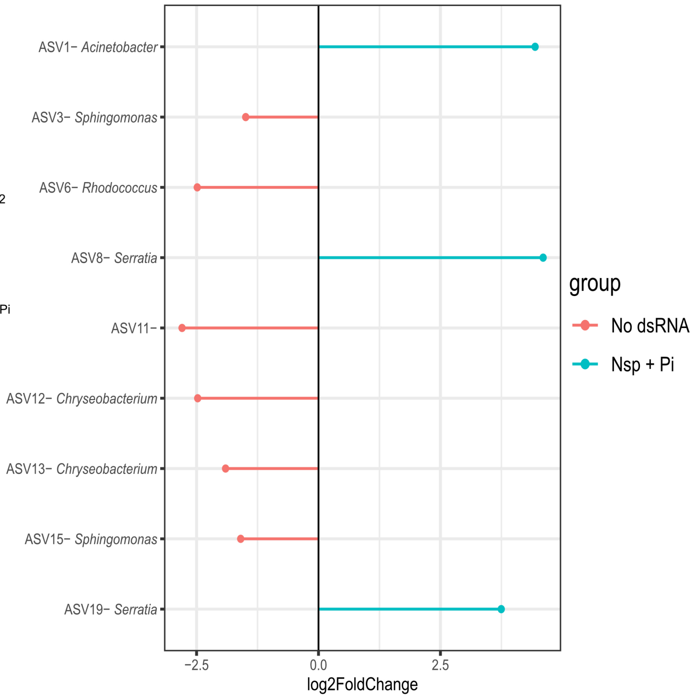

S2. Fungal beta-diversity

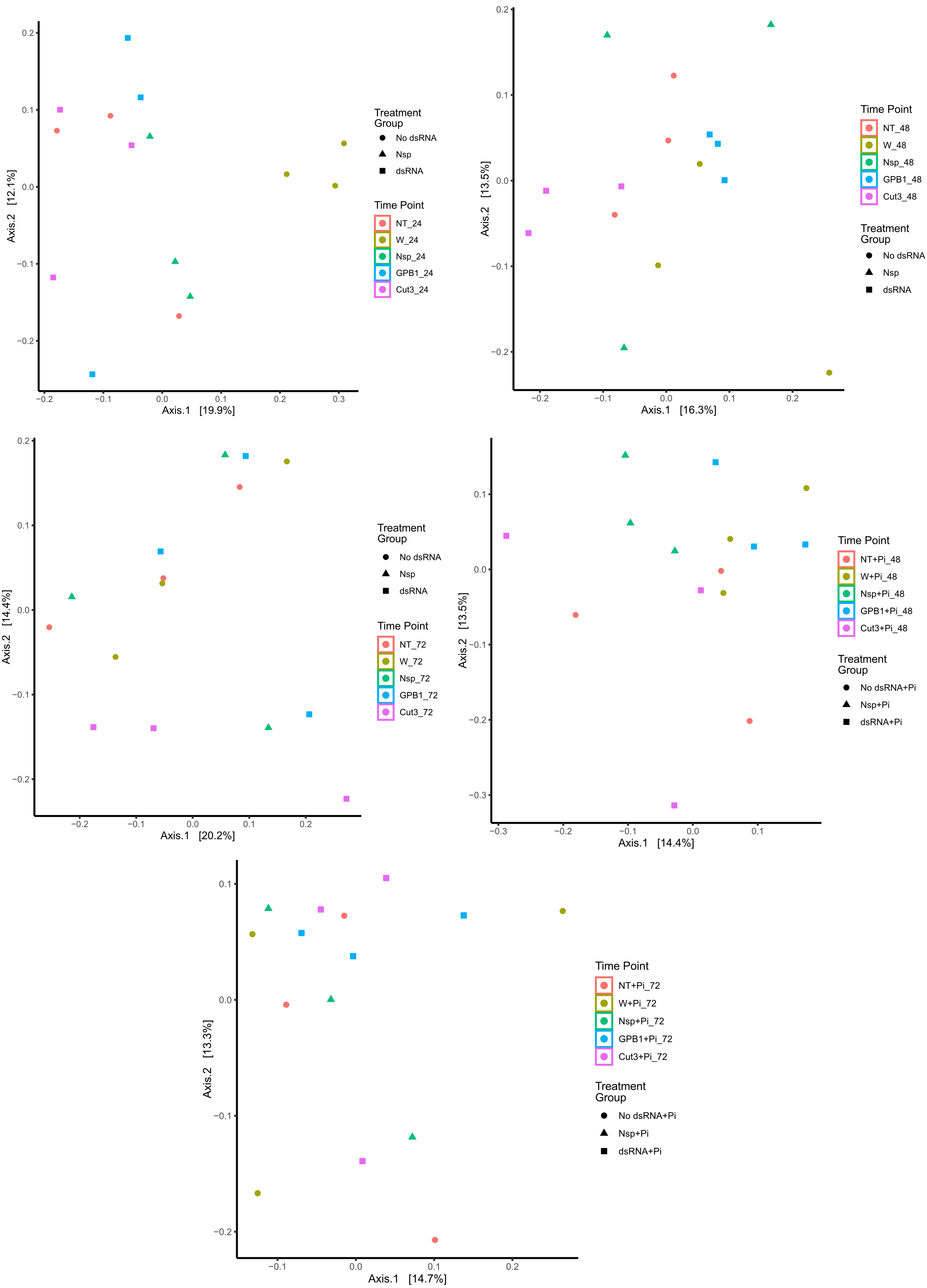

Supplementary Figure 1: Additional bacterial community characteristics. a, PCoA plots based on Bray-Curtis dissimilarity, showing the shifts in bacterial community between treatments at each sampling time. Colours indicate treatments and shapes indicate treatment groups. **b,** Differentially abundant ASVs in Nsp + Pi relative to ND detected using ANCOM-BC. All ASVs shown in the plot have significantly different fold changes ($q < 0.05$). Blue lines represent enrichment and orange lines represent depletion.

Supplementary Figure 2: Fungal beta-diversity at individual time points. PCoA plots using Bray-Curtis dissimilarity distance to show the shifts in the fungal communities between treatments. The individual plots show community diversity at each time point sampled. Colours indicate treatment and shapes indicate treatment groups.

Supplementary data S1
Sequence statistics for bacteria, fungi and oomycetes.

Supplementary data S2
Abundances of bacterial, fungal and oomycete amplicon sequence variants (ASVs) across each sample.

Supplementary data S3
Taxonomy of all bacterial, fungal and oomycete ASVs.

Supplementary data S4
Sequences of all bacterial, fungal and oomycete ASVs.

Supplementary data S5
The α-diversity measure (Simpson) values for bacteria and fungi in different treatments and treatment groups.

Supplementary data S6
The beta-diversity (PCoA) analyses for bacteria and fungi comparing different timepoints, treatments and treatment groups.

Supplementary data S7
Composition (relative abundance) analysis of bacterial communities across timepoints, treatments and treatment groups.

Supplementary data S8
Differential abundance (ANCOM-BC) analysis of bacterial and fungal communities between different treatment groups.

Supplementary data S9
Composition (relative abundance) analysis of fungal communities across timepoints, treatments and treatment groups.

Supplementary data S10
Composition (relative abundance) analysis of oomycete communities across timepoints, treatments and treatment groups.

Supplementary data S11
Bacterial and fungal co-occurrence network topologies for no-dsRNA (ND) and dsRNA treatment groups, and keystone species analysis (ZiPi) within groups.

Supplementary data S12
Upset plot values for bacterial and fungal communities across treatments and treatment groups.
